# Genome-wide identification and expression analysis of *VQ* gene family under abiotic stress in *Coix lacryma-jobi* L.

**DOI:** 10.1186/s12870-023-04294-9

**Published:** 2023-06-20

**Authors:** Yujiao Wang, Xianyong Lu, Yuhua Fu, Hongjuan Wang, Chun Yu, Jiasong Chu, Benli Jiang, Jiabao Zhu

**Affiliations:** 1 Department of Cotton Research Institute, Anhui Academy of Agricultural Sciences, Hefei, 230001 China; 2 Guizhou Institute of Subtropical Crops, Guizhou Academy of Agricultural Sciences, Xingyi, China

**Keywords:** *Coix lacryma-jobi*, Valine glutamine (VQ), Phytohormone, Drought stress

## Abstract

**Background:**

Valine-glutamine (VQ) proteins are non-specific plant proteins that have a highly conserved motif: FxxhVQxhTG. These proteins are involved in the development of various plant organs such as seeds, hypocotyls, flowers, leaves and also play a role in response to salt, drought and cold stresses. Despite their importance, there is limited information available on the evolutionary and structural characteristics of *VQ* family genes in *Coix lacryma-jobi*.

**Results:**

In this study, a total of 31 *VQ* genes were identified from the coix genome and classified into seven subgroups (I–VII) based on phylogenetic analysis. These genes were found to be unevenly distributed on 10 chromosomes. Gene structure analysis revealed that these genes had a similar type of structure within each subfamily. Moreover, 27 of *ClVQ* genes were found to have no introns. Conserved domain and multiple sequence alignment analysis revealed the presence of a highly conserved sequences in the ClVQ protein. This research utilized quantitative real-time PCR (qRT-PCR) and promoter analysis to investigate the expression of *ClVQ* genes under different stress conditions. Results showed that most *ClVQ* genes responded to polyethylene glycol, heat treatment, salt, abscisic acid and methyl jasmonate treatment with varying degrees of expression. Furthermore, some *ClVQ* genes exhibited significant correlation in expression changes under abiotic stress, indicating that these genes may act synergistically in response to adversarial stress. Additionally, yeast dihybrid verification revealed an interaction between ClVQ4, ClVQ12, and ClVQ26.

**Conclusions:**

This study conducted a genome-wide analysis of the *VQ* gene family in coix, including an examination of phylogenetic relationships, conserved domains, cis-elements and expression patterns. The goal of the study was to identify potential drought resistance candidate genes, providing a theoretical foundation for molecular resistance breeding.

**Supplementary Information:**

The online version contains supplementary material available at 10.1186/s12870-023-04294-9.

## Introduction

*Coix lacryma-jobi,* commonly referred to as Adlay or Job's tears, is a perennial herbaceous plant belonging to the Poaceae family. It is widely cultivated in East and Southeast Asian countries for its nutritional and therapeutic properties [1]. The plant's grain is a rich source of protein, making it the most protein-rich cereal crop. Additionally, extracts from its seeds are used to treat various ailments, further highlighting its medicinal value [2, 3]. Coix is primarily cultivated, produced, and consumed in China where it has been grown for over 6000 years [4]. Guizhou Province, in particular, has emerged as the primary production region for coix in China and Southeast Asia, with a cultivation area of 50,000 hm2 as of 2021 [5].

The VQ protein family is a vital plant transcription regulatory cofactor that plays a crucial role in controlling plant development, growth, and responses to environmental stresses [6]. VQ conserved domain can be categorized based on differences in the last three amino acids of the VQ domain since members of the VQ gene family all have the same core conserved sequence (FxxhVQxhTG) [7, 8]. For instance, grape has only three varieties (LTG, FTG and VTG) while Arabidopsis have six (LTG, FTG, VTG, YTG, LTS and LTD) [9, 10]. Additionally, the majority of VQ genes lack introns and the length of protein sequences are less than 300 aa.

The *VQ* genes perform a variety of roles during various stages of plant growth and development, including organ development, biotic and abiotic stress response, and defense response [6]. In Arabidopsis, *AtVQ20* is expressed specifically in pollen and interacts with *AtWRKY2* and *AtWRKY34* to regulate pollen development [11]. *AtVQ18* and *AtVQ26* are negative interacting factors of ABI5 transcription factors, which fine-tune seed germination by antagonizing *ABI5* to maintain appropriate ABA signaling levels [12]. Recent research has shown that *WRKY75* and *SIBs* could collaborate to control ABA-mediated leaf senescence and seed germination [13]. It was found that *OsVQ25* plays a crucial role in maintaining a balance between disease resistance and plant growth through the interaction of *OsVQ25* with *OsPUB73* and *OsWRKY53* [14]. Numerous studies have found that the expression of *VQ* gene is induced by salt, drought, and temperature stresses as well as ABA [6, 15, 16]. The expression of most genes in cotton, maize and rice was induced under drought, salt, cold stress, and heat stress [17–19]. Overexpression of *PeVQ28* enhances salt tolerance in Arabidopsis by reducing malondialdehyde content and increasing proline content [20]. *IbWRKY2* has been found to enhance drought and salt tolerance and interacts with *IbVQ4*. Additionally, it has been observed that PEG and NaCl treatments lead to a similar increase in *IbVQ4* expression, suggesting that *IbVQ4* may be an essential factor in sweet potato's ability to tolerate drought and salt stress [21]. The data clearly showed that overexpression of *MdVQ37* decreased the tolerance of transgenic apple lines to heat and drought stress [22]. VQ proteins act as transcriptional regulatory cofactors and play a crucial role in regulating various physiological and biochemical processes in plants [23–25]. Among the interacting proteins of VQ, WRKY transcription factors are the most significant [26–28]. Additionally, VQ proteins can also interact with each other, as observed by Wang et al. For instance, *AtVQ12* can strongly interact with *AtVQ3*, *AtVQ8*, *AtVQ10*, *AtVQ12*, *AtVQ17*, *AtVQ18*, *AtVQ29*, and *AtVQ32* [22].

The VQ protein family has been identified in several species, but little is known about its members in coix. However, with the complete coix genome now available, researchers have the opportunity to conduct a thorough investigation of *VQ* genes in coix. This study aims to identify VQ protein family members from the coix genome and analyze their phylogenetic relationships, gene structure, and conserved motifs using bioinformatics tools. Further, chromosome distribution and cis-elements were analyzed. Finally, the expression levels of *VQ* genes in different tissues and in response to stresses (ABA, MeJA, drought) were analyzed. In addition, the coregulatory networks of *ClVQs* under abiotic stress were analyzed based on the PCCs of their relative expression levels. The detailed information provided in this study will contribute to further understanding of the VQ gene family. Meanwhile, the basis for further research on the biological functions of VQ genes and screening of candidate genes for resistance in coix.

## Results

### A total of 31 VQ genes were identified in coix

An HMM search was performed against the coix genome database, a total of 40 VQ-containing sequences were obtained. After manual de-duplication, a total of 31 nonredundant *VQ* genes were identified, which were named *ClVQ1* to *ClVQ31* according to their physical location (from top to bottom) on chromosome. In Table 1, these genes encode proteins ranging from 85 to 1408 amino acids (aa), with the majority of ClVQ proteins being less than 300aa in length (87.1% of coix). The molecular weight ranged from 9353.38 Da (*ClVQ31*) to 155310.57 Da (*ClVQ18*), and the predicted isoelectric point was 5.06 (*ClVQ12*) to 10.97 (*ClVQ19*). Analysis of the cellular localization of ClVQ proteins showed that most ClVQ proteins were localized in the nucleus, some in the chloroplast, and two ClVQ proteins were localized in the mitochondria (Table S1). Additionally, a three-dimensional model of the ClVQ proteins was created using a Swiss-Model web server (Fig. S1). Most homologous pairs of the protein have different three-dimensional structures, suggesting potential functional variety.

**Table 1:** Details of the identified *ClVQ* genes

**Name**	**Gene Identifier**	**Location**	**ORF length (bp)**	**Protein**			
				**Length (a.a.)**	**PI**	**Mol.Wt. (Da)**	**Exons**
*ClVQ1*	Cl017671_T1	Chr1:10323082..10323943	705	234	10.4	23771.31	2
*ClVQ2*	Cl019380_T1	Chr1:49425867..49426730	864	287	8.82	30137.97	1
*ClVQ3*	Cl020396_T1	Chr1:156501734..156502135	402	133	6.82	14353.06	1
*ClVQ4*	Cl000473_T1	Chr2:6309585..6310178	594	197	6.43	20985.22	1
*ClVQ5*	Cl000656_T1	Chr2:8951166..8951897	732	243	10.9	25216.57	1
*ClVQ6*	Cl001487_T1	Chr2:21666176..21666712	537	178	8.07	18197.02	1
*ClVQ7*	Cl001606_T1	Chr2:24792544..24793188	645	214	7.84	21857.68	1
*ClVQ8*	Cl002136_T1	Chr2:34913778..34914668	891	296	9.05	30245.5	1
*ClVQ9*	Cl002879_T1	Chr2:58387199..58387981	783	260	7.88	26524.77	1
*ClVQ10*	Cl005732_T1	Chr2:176638900..176639427	528	175	11	18163.87	1
*ClVQ11*	Cl026223_T1	Chr3:12773846..12774622	777	258	7.35	26181.84	1
*ClVQ12*	Cl026639_T1	Chr3:32524660..32525238	579	192	5.06	20878.53	1
*ClVQ13*	Cl026809_T1	Chr3:44946616..44947272	657	218	10.5	22032.86	1
*ClVQ14*	Cl027282_T1	Chr3:91338266..91338640	375	124	7.82	13364.03	1
*ClVQ15*	Cl006467_T1	Chr4:1557742..1558497	756	251	6.33	25881.22	1
*ClVQ16*	Cl006939_T1	Chr4:7821656..7822342	687	228	6.86	23254.79	1
*ClVQ17*	Cl009284_T1	Chr4:58154166..58155515	1350	449	6.48	44285.14	1
*ClVQ18*	Cl010380_T1	Chr4:139422371..139433003	4227	1408	7.45	155310.6	9
*ClVQ19*	Cl029546_T1	Chr5:5018347..5019111	765	254	11	26171.85	1
*ClVQ20*	Cl029762_T1	Chr5:8673329..8673864	507	168	4.44	16842.47	2
*ClVQ21*	Cl013179_T1	Chr6:19907346..19914547	882	293	7.86	31082.46	2
*ClVQ22*	Cl014167_T1	Chr6:37112888..37113145	258	85	9.1	9390.39	1
*ClVQ23*	Cl033684_T1	Chr7:43528212..43529414	1203	400	7.84	40598.52	1
*ClVQ24*	Cl038325_T1	Chr8:141599917..141600591	675	224	9.13	23249.17	1
*ClVQ25*	Cl038326_T1	Chr8:141623530..141624261	732	243	9.29	24671.47	1
*ClVQ26*	Cl021872_T1	Chr9:1099634..1100230	597	198	10.3	21147.02	1
*ClVQ27*	Cl022088_T1	Chr9:3558783..3559259	477	158	6.51	16369.4	1
*ClVQ28*	Cl022372_T1	Chr9:7243171..7243860	690	229	9.51	24141.31	1
*ClVQ29*	Cl041459_T1	Chr10:133307856..133308575	720	239	10.1	24140.92	1
*ClVQ30*	Cl043095_T1	Unplaced_contig5:752346..753404	1059	352	7.26	35749.07	1
*ClVQ31*	Cl042147_T1	Unplaced_contig315:31568..31840	273	90	9.22	9353.38	1

### Phylogenetic tree of VQ domains in Arabidopsis, rice, maize, and coix

**Fig. 1:**
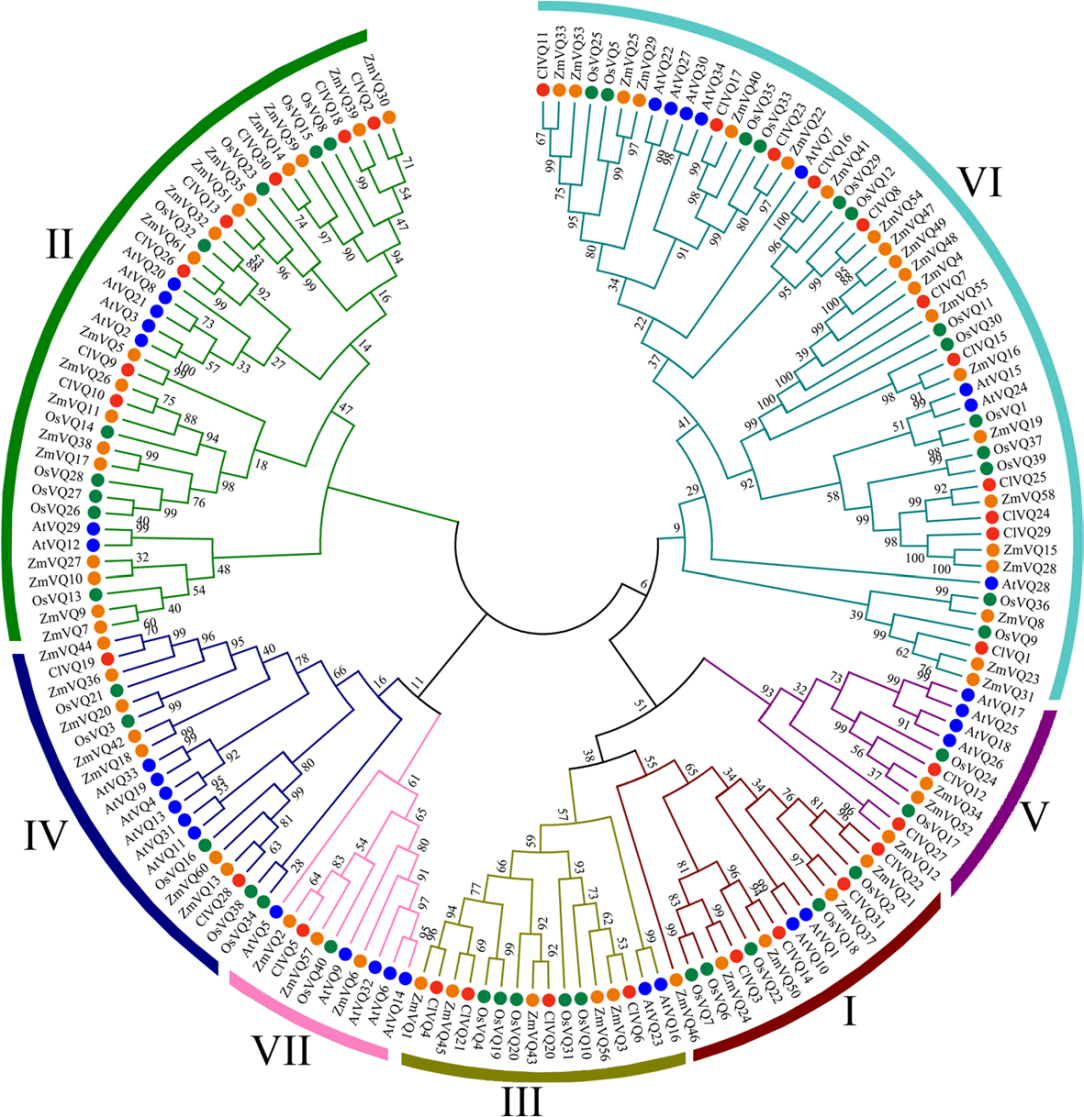
Phylogenetic tree of *VQ* genes from coix, Arabidopsis, rice and maize. 31 *ClVQ* genes, 34 *AtVQ* genes, 40 *OsVQ* genes and 61 *ZmVQ* genes are clustered into 7 subgroups (I-VII). *VQ* genes from coix, Arabidopsis, rice and maize are denote by red, blue, green and yellow shape, respectively. Details of the *VQ* genes from four species are listed in Table S2. The tree was generated with the Clustal X 2.0 software using the neighbor-joining (N-J) method

To understand the evolutionary relationships of VQ genes, a phylogenetic tree was constructed using 166 VQ proteins from Arabidopsis, rice, maize and coix (Fig. 1). Also, Table 1 and Table S2 contain comprehensive information about VQ genes. The 31 *ClVQ* genes were classified into seven subgroups (I-VII) based on the classification of Arabidopsis, rice, and maize *VQ* gene families. Subgroup VI had the highest number of members (11), followed by subgroup II (7), while subgroup VII had the lowest number of members with only one. This distribution pattern was similar to that of *VQ* genes in rice and maize [17, 18]. Moreover, coix, rice, and maize all belong to the gramineae family, resulting in a relatively even distribution of *VQ* genes among the phylogenetic tree of these three species.

### Conserved motifs, multiple alignment, and gene structural analysis

**Fig. 2:**
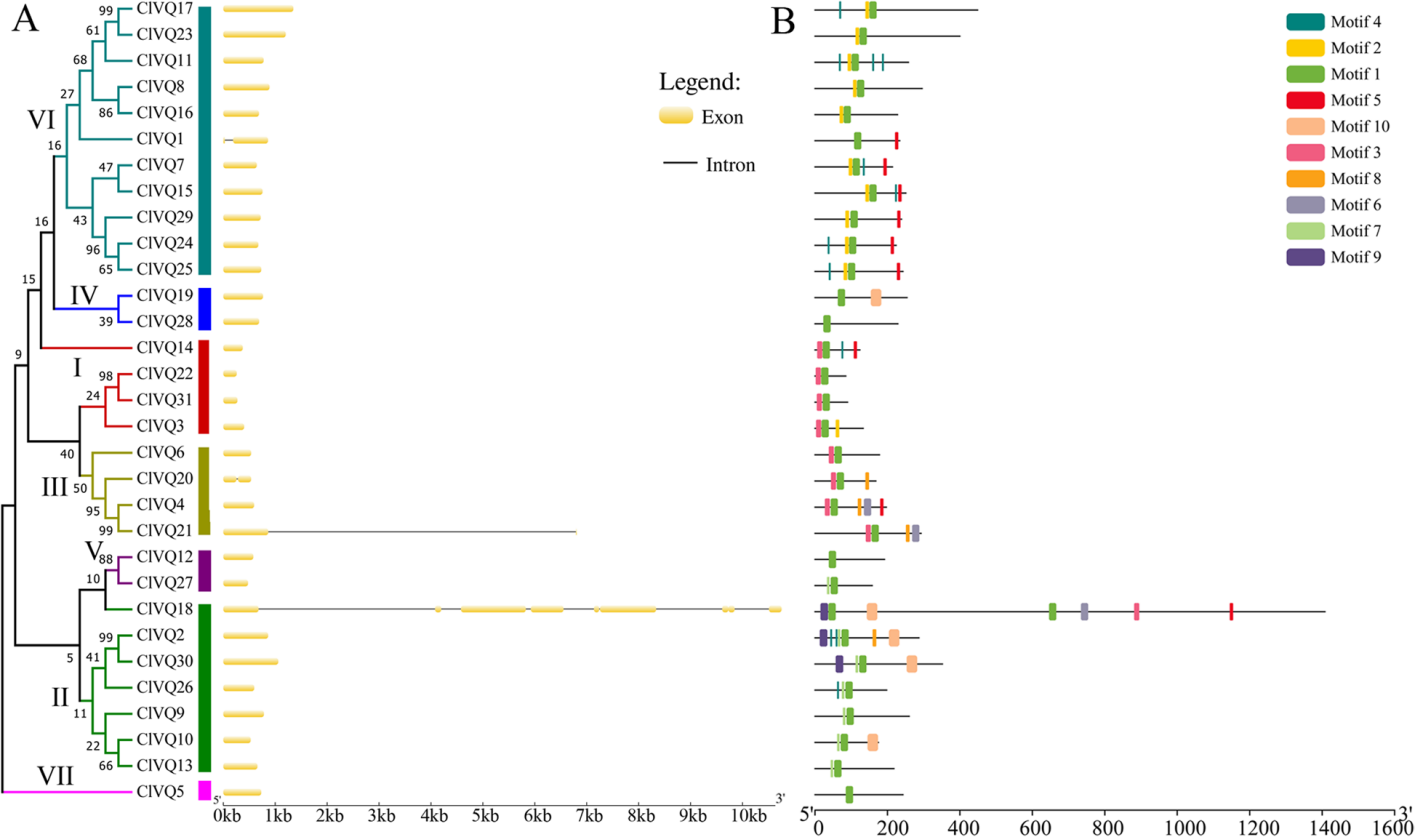
Phylogenetic relationships, gene structure and Conserved motifs of *VQ* genes in coix. **A** Phylogenetic relationships and gene structure of VQ genes in coix. Exons are indicated by yellow rectangles. Gray lines connecting two exons represent introns. **B** Conserved motifs of VQ genes in coix. Distribution of the 10 conserved motifs in the *ClVQ* genes following analysis by MEME tool. The different-colored boxes represent different motifs and their position in each protein sequence of ClVQ

Exon/intron structures were created based on the coding sequences of each *ClVQ* gene to gain a better understanding of the structural diversity of *ClVQ* genes. As shown in Fig. 2A, subgroup I members had shorter coding regions ranging from 273 bp to 402 bp compared to the other subgroups. The results revealed that 27 out of 31 *ClVQ* genes (87.1%) had no introns, which was consistent with previous studies on *AtVQs*, *ZmVQs*, and *OsVQs*. Only *ClVQ1*, *ClVQ18*, *ClVQ20*, and *ClVQ21* were found to contain introns. In particular, the number of introns in *ClVQ18* was much higher than in other genes. Similarly, there is one such gene in Chinese cabbage and Moso bamboo [29, 30].

The conserved motifs in VQ proteins of coix were analyzed by Motif Elicitation tools. Table S3 displays the length and conserved amino acid sequences of the 10 unique motifs that were discovered. The potential motif sequences identified from MEME were annotated by scanning Pfam. The study found that Motif 1 encodes the VQ domain, while the other motifs lacked functional annotation. Fig. 2B illustrates that each ClVQ protein had 1-6 conserved motifs, with Motif 1 present in all VQ proteins. Subgroup VII members contained both Motif 1 and 2, while subgroup III members contained both Motif 1 and 3. The results indicated that VQ proteins within the same subgroup exhibited similar motifs, which aligned with the findings of phylogenetic analysis.

**Fig. 3:**
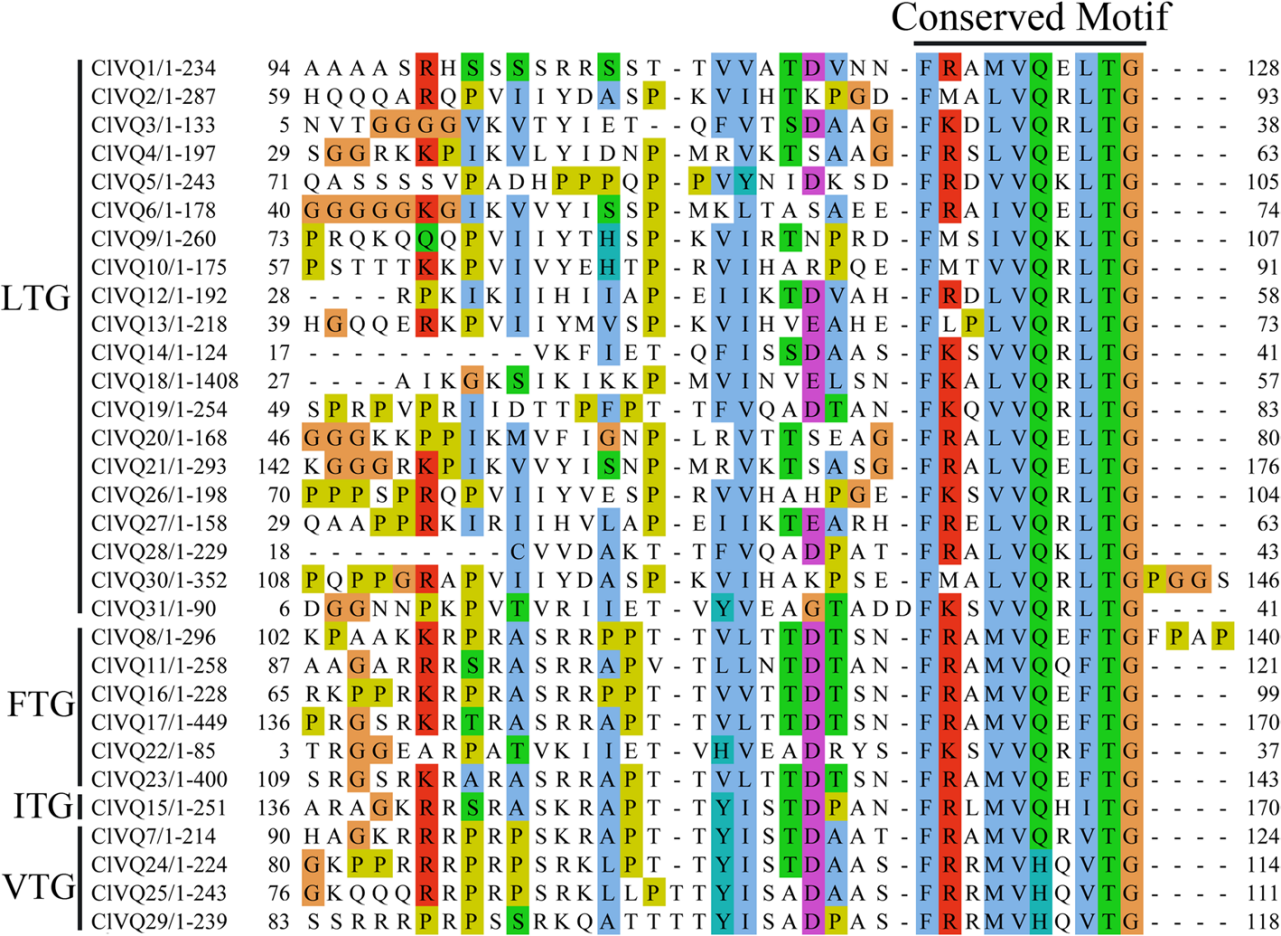
Multiple sequence alignment of *VQ* genes in coix. Sequences were aligned using Jalview software

To better understand the characteristics of coix VQ domain, a multiple sequence alignment was constructed. As depicted in Fig. 3, ClVQ proteins were found to have four types of VQ domains: FxxxVQxLTG (20/31), FxxxVQxFTG (6/31), FxxxVQxITG (1/31), and FxxxVQ/HxVTG (4/31). In contrast to AtVQs and ZmVQs, ClVQ proteins lacked the FxxxVQxLTD, FxxxVQxATG, and FxxxVQxYTG domains [9, 18].

### Chromosomal location and gene pairs analysis in coix

**Fig. 4:**
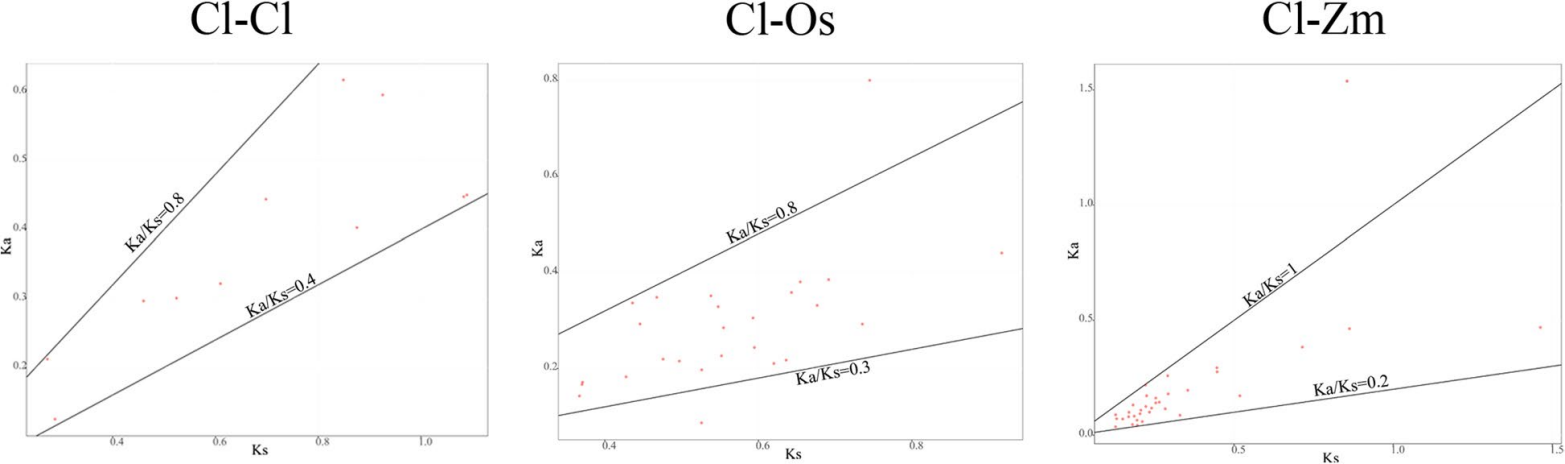
Ka/Ks ratios of paralogs and orthologs. The black lines indicated Ka/Ks equal to 0.2, 0.3, 0.4, 0.8 and 1. The y-axis indicates the value of Ka and the x-axis indicates the value of Ks

Based on the coix database, there are 29 *ClVQ* genes located unevenly across 10 chromosomes, with the exception of *ClVQ30* and *ClVQ31* (Fig. S2). Chromosome 2 contains the highest number of *ClVQ* genes with 7, while chromosomes 7 and 10 have the lowest number with only 1. The remaining chromosomes have 2-4 *ClVQ* genes. Additionally, 11 paralogues were identified in coix using BLASTN methods (Table S4). We found 26 orthologues between *ClVQ* and *OsVQ* genes, and 35 orthologues between *ClVQ* and *ZmVQ* genes. We calculated Ks values, Ka values, and Ka/Ks ratios of both paralogues and orthologues to examine the influence of selection pressure on the evolution of the *ClVQ* gene family (Table S4 and Fig. 4). In general, Ka/Ks ratios below 1, larger than 1, and equal to 1 suggest purifying selection, positive selection, and neutral selection, respectively. The Ka/Ks ratio for all paralogues ranged from 0.4 to 0.8, while for most orthologues it was between 0.2 and 1.0 (Fig. 4). These findings suggest that purifying selection may have played a significant role in the evolution of *VQ* genes in coix.

### Identification of cis-elements in the promoter regions of *ClVQs*

**Fig. 5:**
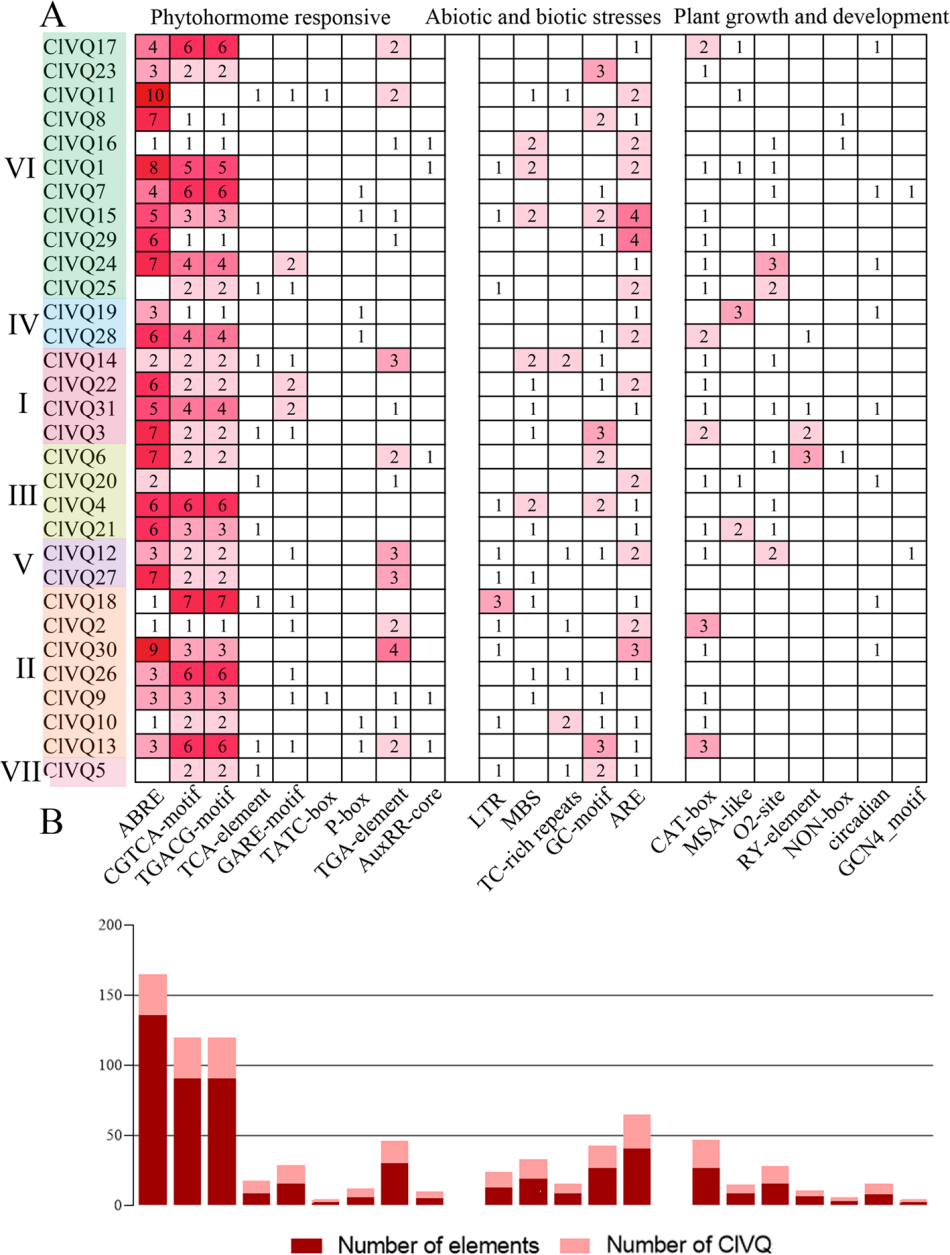
Cis-acting elements analysis of *VQ* genes in promoter region of coix. **A** Number of each cis-acting element in the promoter region (2000 bp) of *ClVQs*. **B** Statistics for the total number of *ClVQs*

To investigate the regulatory mechanism of the *ClVQ* gene, we conducted cis-acting element analysis on a 2000bp sequence of its promoter region. Our analysis also revealed the presence of 386 hormone response elements, 109 stress-related response elements, and 72 growth and development elements in the promoter sequences of 31 *VQ* genes. Fig. 5 shows that most *ClVQ* genes are involved in the ABA signaling pathway, with 136 ABA-responsive elements (ABRE) found in the promoters of 29 *ClVQs* (excluding *ClVQ25* and *ClVQ5*). The promoters of these 29 ClVQs also contain MeJA-responsive elements (CGTCA-motif and TGACG-motif), as well as SA-responsive elements (TCA-element) and gibberellin-responsive elements (P-box, TATC-box and GARE-motif), with 9 and 24 of each, respectively, found in the promoter region of the gene. In the promoters of 14, 11, 25, and 7 *ClVQs*, various cis-elements related to abiotic and biotic stresses were identified. These include MBS (drought induced response element), LTR (low temperature response element), ARE (anaerobic induced response element), and TC-rich (defense and stress response element). In addition, 72 elements related to plant growth and development were identified in the promoter regions. Among these, 27 elements (CAT-box) were found in the promoters of 20 *ClVQs*, which are associated with meristem expression.

### Expression pattern of the *ClVQs* in different tissues

**Fig.6:**
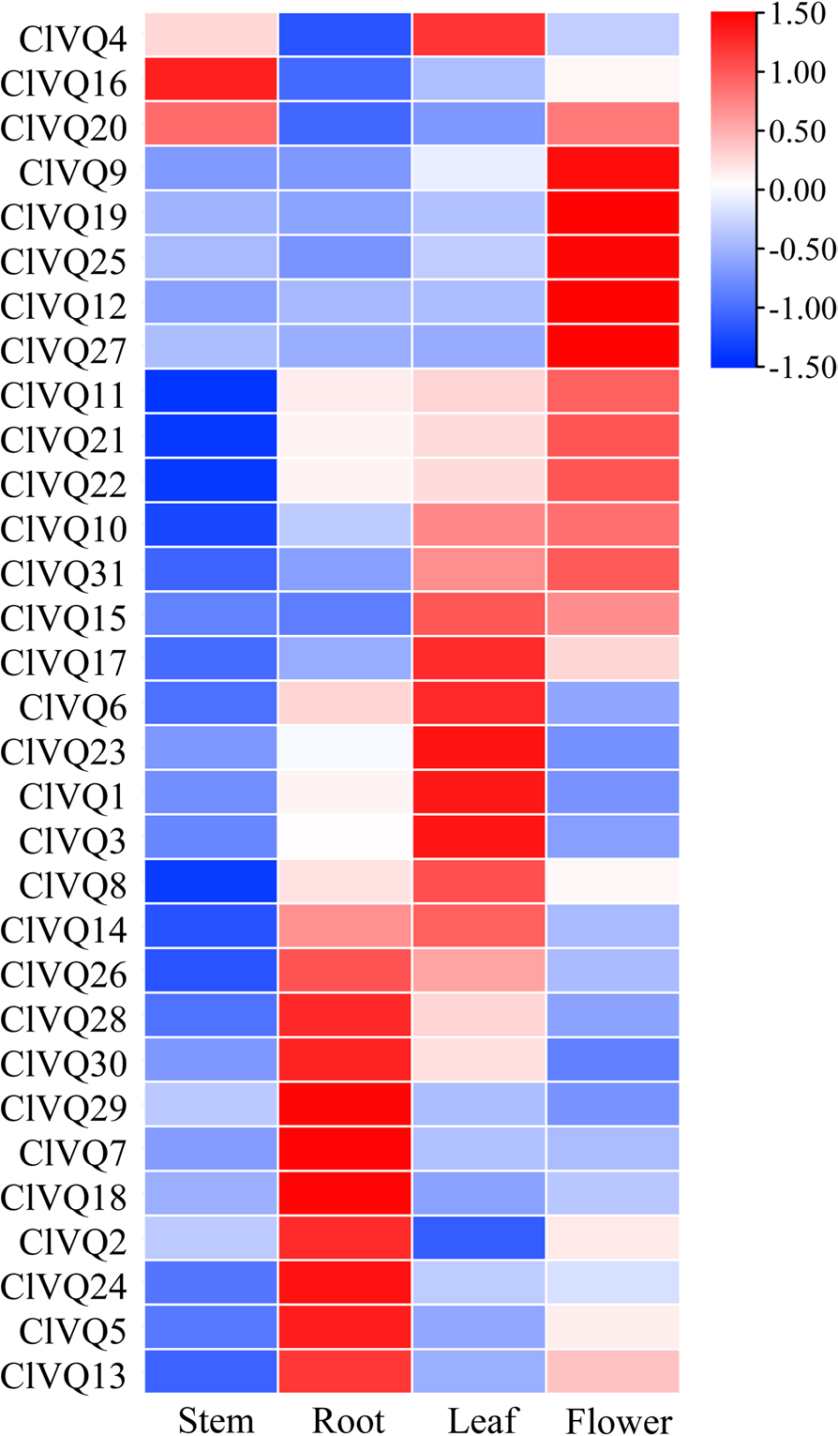
Tissue-specific expression patterns of 31 *ClVQ* genes in coix by qRT-PCR. The heatmap shows the hierarchical clustering of 31 ClVQ genes at different time points. The color scale: Blue represents low expression and red indicates a high expression level

The expression patterns of *ClVQ* genes were analyzed in root, stem, leaf, and flower using qRT-PCR. As shown in Fig. 6, the expression patterns of various *VQ* genes differed among tissues, with members of subgroups III and VI exhibiting high expression in all tested tissues. Over 50% of *ClVQ* genes were up-regulated in leaves, while 9 and 5 *ClVQ* genes were found to be highly expressed in roots and flowers, respectively.The study found that out of 11 paralogous genes, 6 had similar expression patterns and were highly expressed in the same tissue. For instance, *ClVQ29/ClVQ24* and *ClVQ2/ClVQ30* were up-regulated in the root. Moreover, *ClVQ11*, *ClVQ21*, and *ClVQ22* showed increased expression levels in roots, leaves, and flowers, but decreased expression in stems.

### *ClVQ* genes expression following various stresses

**Fig. 7:**
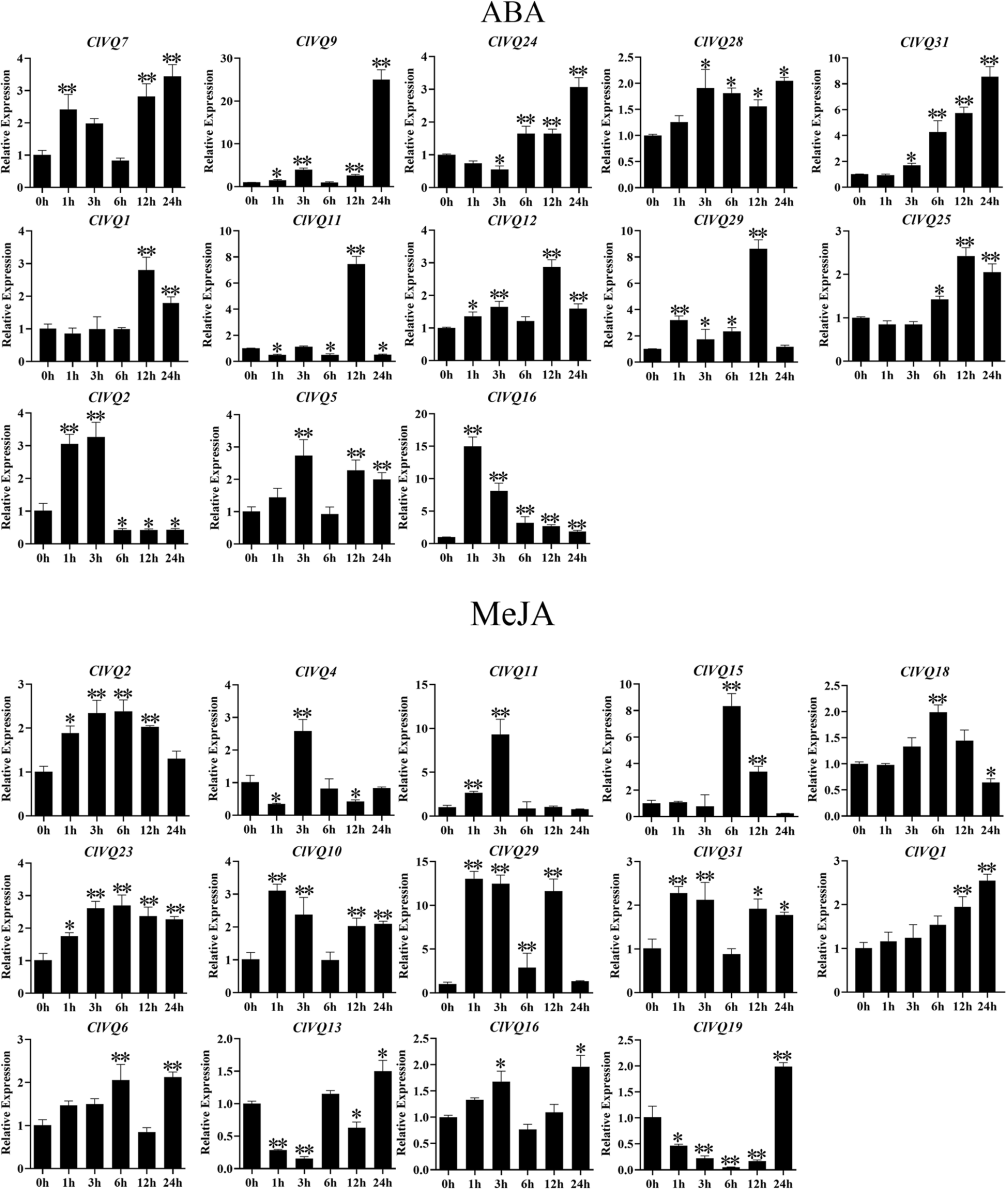
Expression analysis of *ClVQ* genes following hormone treatments by qRT-PCR. **A** Expression patterns of 13 *ClVQs* under ABA treatment. **B** Expression patterns of 15 *ClVQs* under MeJA treatment.The Y-axis and X-axis indicates relative expression levels and the time courses of stress treatments, respectively. Mean values and standard deviations (SDs) were obtained from three biological and three technical replicates. The error bars indicate standard deviation

Promoter analysis revealed that *ClVQ* genes play a crucial role in hormone response. To investigate the effects of hormone treatment on the expression pattern of *ClVQ* genes, the expression levels of 31 *ClVQ* genes were quantified using qRT-PCR following ABA and MeJA administration. The study found that treatment with ABA resulted in the up-regulation of 24 out of the 29 *ClVQ* genes studied, as shown in Fig. S3. Notably, *ClVQ7*, *ClVQ9*, *ClVQ24*, *ClVQ28*, and *ClVQ31* exhibited significant increases in expression levels, with *ClVQ9* showing a particularly high increase of over 20 times that of the control group (Fig. 7). However, the expression levels of *ClVQ17*, *ClVQ18*, and *ClVQ30* did not show significant changes. Three *ClVQ* genes (*ClVQ2*, *5*, and *16*) were significantly up-regulated at early time points, but their expression levels decreased later on. On the other hand, expression of *ClVQ4*, *ClVQ6*, *ClVQ15* and *ClVQ27* was down-regulated during ABA treatment, with *ClVQ4* being consistently suppressed. These results suggest that ABA treatment has a selective effect on the expression of *ClVQ* genes.

After treatment with MeJA, the expression of 31 *ClVQ* genes showed changes when compared to the untreated control (Fig. S4). Among these genes, *ClVQ2*, *4*, *11*, *15*, *18* and 23 exhibited similar expression patterns, as depicted in Fig. 7. For instance, *ClVQ15* was upregulated and reached its peak at 6 h, followed by a decline. During MeJA treatment, *ClVQ23* and *ClVQ29* showed consistent up-regulation at all time points, while *ClVQ7*, *ClVQ21*, and *ClVQ24* were consistently down-regulated. *ClVQ13* and *ClVQ19* were significantly down-regulated at early time points (1 h, 3 h, 6 h, and 12 h), but showed a considerable 1.5-fold up-regulation at 24 h, which differs from the expression patterns of other *VQ* genes.

**Fig. 8:**
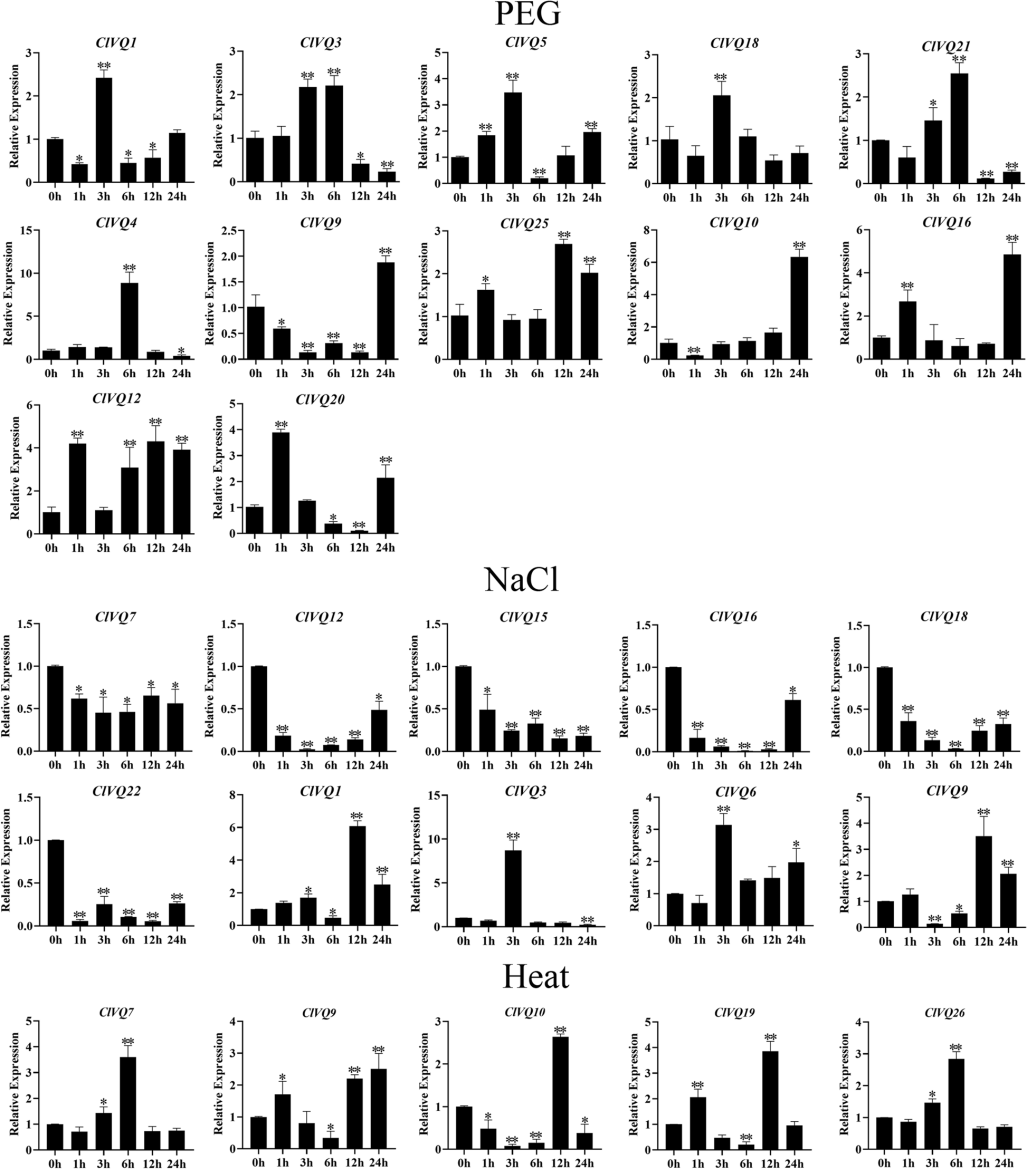
Expression analysis of 20 *ClVQ* genes following drought treatments by qRT-PCR. The Y-axis and X-axis indicates relative expression levels and the time courses of stress treatments, respectively. Mean values and standard deviations (SDs) were obtained from three biological and three technical replicates. The error bars indicate standard deviation

This study aimed to investigate the impact of adverse environmental conditions on the growth and development of plants, specifically focusing on the expression of *ClVQ* genes. The study utilized qRT-PCR to analyze the expression patterns of these genes under drought, salt, and heat stress (Figs. S5, S6 and S7). After drought treatment (Fig. 8), it was observed that the expressions of 14 *ClVQ* genes were up-regulated at varying time intervals. Specifically, after 24 hours of drought treatment, the expression levels of eight *ClVQ* genes were found to be up-regulated. Notably, the expression levels of *ClVQ10* and *ClVQ16* were significantly up-regulated to 6-fold and 4-fold of the control after 24 hours, respectively. During salt stress (Fig. 8), it was found that the six *VQ* genes (*ClVQ7*, *ClVQ12*, *ClVQ15*, *ClVQ16*, *ClVQ18*, and *ClVQ22*) were consisitently down-regulated at all times.Conversely, *ClVQ1*, *ClVQ3*, *ClVQ6*, and *ClVQ9* showed obvious upregulation during stress periods. Furthermore, certain genes such as *ClVQ8* and *ClVQ30* exhibited consistent expression levels throughout the duration of the stress. As for heat stress, 15 of the 31 *ClVQ* genes were down-regulated apparently at any time, while *ClVQ6* and *ClVQ23* were also express stably. Specifically, expression of *ClVQ7, ClVQ9, ClVQ10, ClVQ19* and *ClVQ26* were strongly up-regulated more than twofold during heat stress.

### Coregulatory networks of *ClVQs* under abiotic stress

**Fig. 9:**
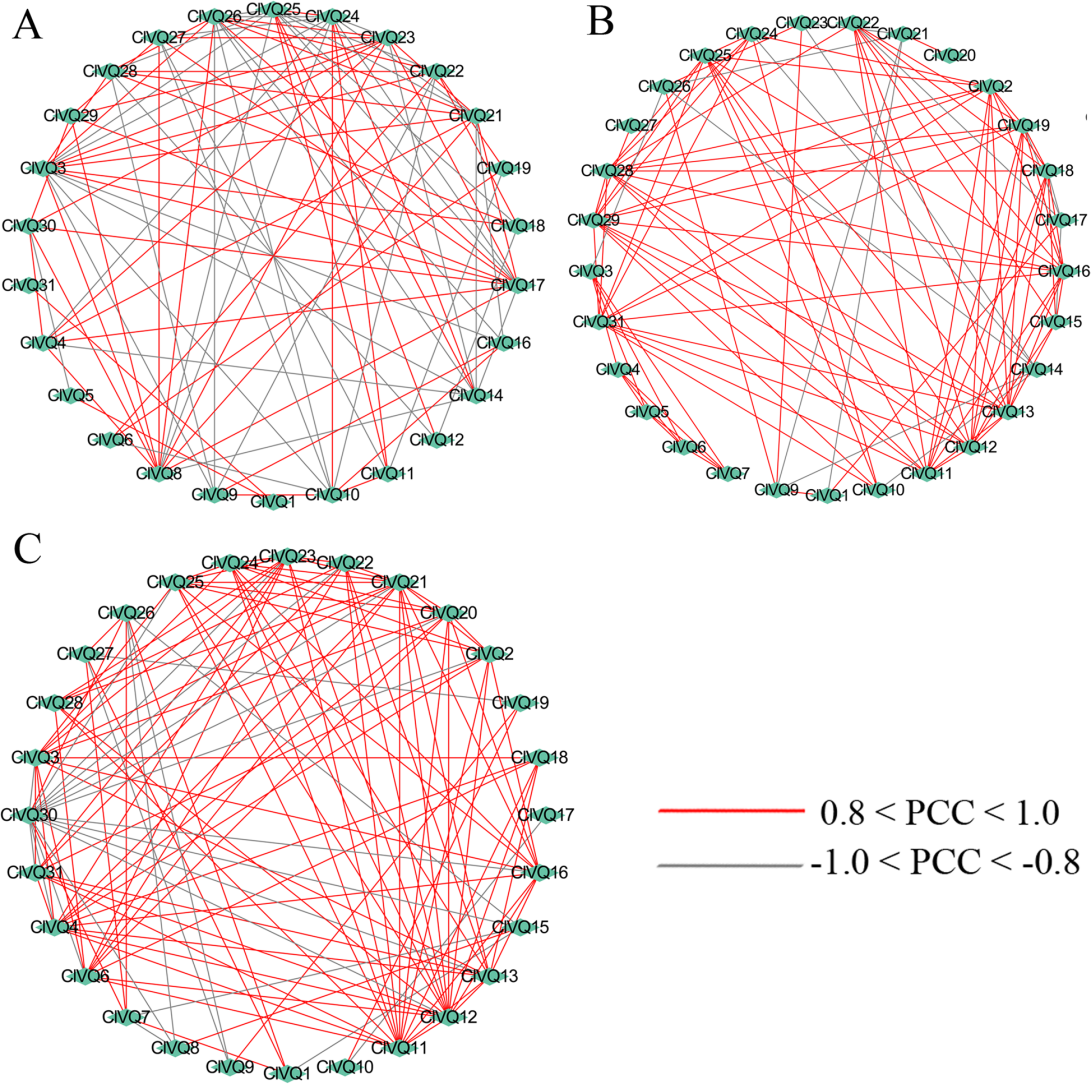
Correlations among *ClVQ* genes under stress treatment. **A** The co-regulatory networks of *ClVQ* genes under PEG treatment. **B** The co-regulatory networks of *ClVQ* genes under NaCl treatment. **C** The co-regulatory networks of *ClVQ* genes under heat treatment were established based on the Pearson correlation coefficients (PCCs) of these gene pairs using transformed qPCR data

To investigate the relationships among genes in response to PEG, NaCl, and heat treatment, correlation and coregulatory networks were established based on the PCCs of their relative expression levels. The coregulatory network was created by gathering and displaying 31 *ClVQ* genes with PCC absolute values larger than 0.8 and significant at the 0.05 significance level (Tables S6 and S7). All *ClVQ* genes appeared to be correlated with each other to varying degrees of positive or negative correlation (Fig. 9). Most *ClVQs* had positive significant correlations between them and were greater than 0.8 under these stresses. The paralogues (*ClVQ24*/*-25*) had positive correlations under three treatment, and *ClVQ26*/*-14 ClVQ14*/*-23* and *ClVQ16*/*-17* had negative correlations under PEG and NaCl treatment.

### Physical interaction between VQs

**Fig. 10:**
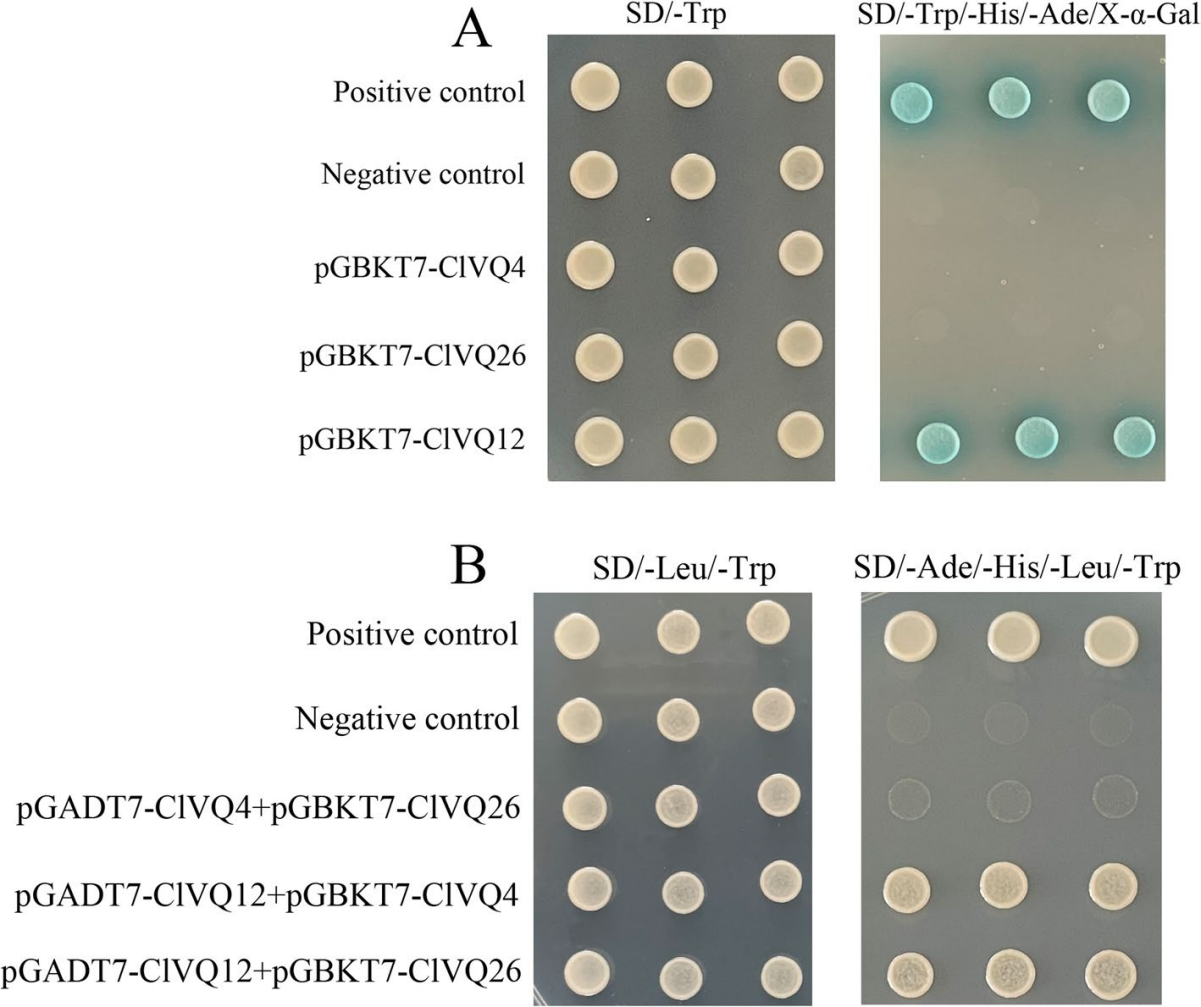
Physical interaction between VQs. **A** Transcriptional activation validation assays. **B** Yeast-two-hybrid assays. ClVQ12, ClVQ4, and ClVQ26 can interact with each other. Sequences of full-length ClVQ4, ClVQ12 and ClVQ26 were fused to the pGBKT7 binding domain (BD, bait), sequences of full-length ClVQ4 Aand ClVQ12 were fused to the pGADT7 activation domain (AD, prey)

The results of the experiment showed that the positive control and pGBKT7-ClVQ12 transformants grew normally and turned blue on D/-Ura/-His/-Trp/-Leu/X-α-Gal medium. On the other hand, the negative control, pGBKT7-ClVQ4 transformants, and pGBKT7-ClVQ26 transformants did not grow normally on the plate (Fig. 10A). These findings suggest that ClVQ12 has self-activating activity, whereas ClVQ26 and ClVQ4 do not. Yeast two-hybrid confirmation was performed by observing the growth of transformants from both control and experimental groups on SD/-Trp/-Leu medium. Both positive control and experimental groups grew on SD/-Ura/-His/-Trp/-Leu, while the negative control did not show any growth (Fig. 10B). The results indicate that ClVQ12, ClVQ4, and ClVQ26 can interact with each other, with the interaction between ClVQ4 and ClVQ26 being relatively weaker.

## Discussion

VQ proteins have been identified as transcriptional regulatory cofactors in various plants. They have also been found in fungi, lower animals, and bacteria, with single to several VQ proteins present [31]. Research on VQ gene function has shown that it is not only involved in plant responses to biotic and abiotic stresses but also plays a role in regulating plant growth and developmental processes [12, 16]. However, little is known about *VQ* genes in coix. Therefore, bioinformatic analysis of the *ClVQ* genes and their patterns of expression under various stress treatments may help us better understand the mechanisms that affect plant stress resistance, which could be applied to coix molecular breeding.

A total of 31 *VQ* genes were identified in coix, whereas Arabidopsis had 34 *VQ* genes despite having a much smaller genome size of 135 Mb. This trend was also observed in rice and bamboo, indicating a possible loss of *VQ* genes during genome expansion in these species [17, 30, 32]. A comprehensive phylogenetic tree was used to divide the 31 *ClVQ* genes into seven subgroups. The tree showed that *ClVQs*, *OsVQs*, and *ZmVQs* were consistently clustered together, likely due to the fact that all three species belong to the gramineae family. Additionally, comparative genomics analysis suggested that coix was more closely related to maize than rice [33]. The study found that *ZmVQs* and *ClVQs* had a higher number of orthologous compared to *OsVQs* and *ClVQs* (Table S4). Additionally, the Ka/Ks analysis revealed that *ClVQ* underwent purifying selection. Most ClVQ proteins (87.1%) had an amino acid length of less than 300 aa. Similarly, Arabidopsis, rice, C. pepo, and maize had a high percentage of VQ protein length less than 300 aa, ranging from 81.8% to 90.3% [7, 9, 16, 18]. The subcellular localization of VQ proteins was analyzed and it was found that most ClVQ proteins were present in the nucleus and chloroplasts. A few ClVQ and AtVQ proteins were found in mitochondria (Table S1), while some AtVQ and OsVQ proteins were found in the cytoplasm [17]. These results suggest that the VQ protein may have diverse functions in different cellular locations.The distribution of *ClVQs* on chromosomes was found to be non-uniform. Chromosome 2 contained 7 *ClVQ* genes, while chromosome 10 contained only 1 *ClVQ* gene (Fig. S2). This pattern is consistent with previous studies on *VQ* genes, for instance, Arabidopsis chromosomes 1 to 5 have 11, 7, 8, 4, and 4 *VQ* genes, respectively [9].

In this study, 28 ClVQ proteins were found to contain the conserved FxxxVQxhTG motif, while the other three ClVQ proteins contained the FxxxVHxhTG motif (Fig. 3). The FxxxVHxhTG motif has previously been observed in the VQ gene of maize, rice, and moso bamboo [17, 18, 30]. Further analysis revealed that the conserved domain of 20 ClVQ proteins had LTG as the last three amino acid residues, with only a few members having FTG, ITG, and VTG. This classification based on the differences in the last three amino acids resulted in six types in both Arabidopsis and maize [9, 18]. The VQ family members exhibit functional diversity, and in addition to the highly conserved VQ structural domain, there is also abundant amino acid sequence diversity at other positions. Studies have shown that during long-term evolution, most *VQ* genes have lost introns, which is consistent with the gene structure analysis of 87.1% (27/31) *VQ* genes in coix without introns (Fig. 2).

The promoter sequences of *ClVQ* genes were found to contain several cis-acting elements, with hormone response elements accounting for 68% of them (Fig. 5). The results were similar to wheat and *Brassica juncea* [34, 35], with the highest number of members responding to ABRE elements. This suggests that the expression of most VQ genes is regulated by ABA. *ClVQ11* contained the largest number of ABRE elements, with up to 10 identified. The expression of *ClVQ11* increased to more than 6-fold of the control after ABA treatment for 12 hours. The study also identified several stress-related response elements, as well as growth and development elements. A recent study found that *OsVQ13* positively regulates JA signaling and increases grain size in transgenic rice [36]. *AtVQ8* in Arabidopsis was found to be involved in plant growth and development. The majority of *ClVQs* are expressed in roots and leaves, while five *ClVQs* (*ClVQ9*, *19*, *26*, *12* and *27*) were highly expressed in flowers (Fig. 6). Similarly, almost all *CsVQs* in tea were expressed in the root, stem, and leaf, with four *VQs* highly expressed in flowers of *M. truncatula* [8, 37]. The document demonstrated that *OsVQ1* interacts with *OsMPK6* and enhances the expression of genes that promote flowering [38]. Therefore, it could be concluded that the *ClVQs* is not only involved in hormone signaling and abiotic stress processes, but also plays a crucial role in regulating growth and development.

Recent research has shown that *VQ* genes play a crucial role in responding to different hormones and stresses such as ABA, MeJA, drought, NaCl, and heat. For instance, after ABA or MeJA treatment in wheat, 12 TaVQ genes were found to be induced. In sugarcane, seven genes were found to be affected by JA and ABA treatments [35, 39]. In our study, we found that 29 out of 31 *ClVQs* contained both ABA response elements and MeJA response elements. Additionally, most of these genes were regulated by treatments of ABA or MeJA. Notably, six of these genes (*ClVQ1*, *2*, *5*, *11*, *29* and *31*) were significantly up-regulated after exposure to ABA and SA treatments (Fig. 7). The *VQ* genes is known to have a significant impact on abiotic stress. Overexpression of *PeVQ28* can enhance the salt tolerance of Arabidopsis, while overexpression of *MdVQ37* in transgenic apple can reduce the drought resistance of Arabidopsis [20, 40]. Additionally, the *VQ* genes is sensitive to temperature changes, with the majority of Chinese cabbage *VQ* genes responding to heat [29]. In this work, the expression of *ClVQ* genes (*ClVQ1*, 9, 10 and 27) was significantly up-regulated during NaCl, drought and heat treatment at certain time points. *ClVQ12*, *ClVQ13*, *ClVQ16*, *ClVQ18*, and *ClVQ22* expression was found to be highly up-regulated with drought treatment, but the expression was suppressed under NaCl and heat treatment. Recent studies have shown that AtVQ12 and AtVQ29 have the ability to form both heterodimers and homologous dimers through physical interaction [22]. Additionally, the yeast dihybrid assay has confirmed the interaction of ClVQ4, ClVQ12, and ClVQ26.

## Conclusion

This work identified 31 *VQ* genes in the genome of coix. A systematic bioinformatics analysis of the *VQ* gene family of coix was performed, including phylogenetic relationships, conserved domain, exon-intron structure and so forth. Through the integration of promoter analysis and expression pattern, it was observed that *ClVQ* genes exhibited positive responses to various stressors, including ABA, MeJA, drought, NaCl, and heat. Notably, *ClVQ1*, *ClVQ9*, *ClVQ10*, *ClVQ26*, and *ClVQ29* displayed significant increases in expression levels in response to various abiotic stresses. Additionally, yeast dihybrid verification revealed an interaction between ClVQ4, ClVQ12, and ClVQ26. In a word, the stress response candidate genes of coix were screened in this study, providing a foundation for further research on the function of VQ family members in abiotic stresses.

## Materials and methods

### Plant materials, growth conditions, and stress treatments

The Wanyi 2 variety of coix is extensively cultivated in Anhui, China (Breeding by the cotton research institute of Anhui academy of agricultural). To conduct the experiment, the seeds were planted in pots filled with a mixture of vermiculite and black soil and were grown in a greenhouse at a temperature of 25°C with a light/dark cycle of 16/8 hours. After three weeks of growth, seedlings of uniform size were selected for studying the expression level of the *VQ* gene of coix under stress treatment.

To conduct stress and hormone treatments, we poured a 200 Mm NaCl, a 20% PEG-6000 solution, a 100 µM ABA solution and a 100 µM MeJA solution over the culture medium vermiculite and black soil, respectively. Heat stress treatments were conducted by controlling the temperature in the plant climate incubator at 40±1°C. We harvested plant leaves at 0, 1, 3, 6, 12, and 24 hours after treatment and immediately froze them in liquid nitrogen. Samples of roots, stems, leaves, and inflorescence tissues were collected from coix plants that had been cultivated for at least three months.

### Database search for* VQs* in coix

The whole genome data of coix was downloaded from Coge (https://genomevolution.org/coge/). The VQ domain Hidden Markov Model (HMM) information with the number PF05678 was obtained from the Pfam database (http://pfam.xfam.org/). The Linux version of HMMER software was used to identify the VQ protein. The coix VQ protein sequence was obtained by using HMMER software for identification with the *E*-value set to less than 10^-5 and redundant sequences were removed. The candidate VQ protein sequences underwent verification through the CDD database on the NCBI website. The protein sequence that contained the VQ domain was ultimately retained. The identified ClVQ proteins had their predicted sequence length, molecular weight, isoelectric point, and subcellular localization determined using the ExPasy and PSORT websites.The 3D structure of each ClVQ protein was determined using SWISS-MODEL (https://swissmodel.expasy.org/interactive) [41].

### Phylogenetic analysis and multiple alignment

To align the VQ amino acid sequences, we utilized MEGA 7.0 software and constructed a phylogenetic tree using the neighbor-joining method (NJ) with a Bootstrap value of 1000 and default parameters [42]. We obtained VQ proteins from *A. thaliana*, rice, and maize from Phytozome v13 and aligned them with 31 ClVQ protein sequences using Jalview software.

### Motif prediction and gene structure analysis

To predict the gene structure, we uploaded the GFF file of 31 *ClVQ* genes to the Gene Structure Display Server 2.0 website. We also used the MEME online tool to query the protein domain [43]. The criteria for the MEME analysis included a site distribution of zero or one occurrence per sequence, a maximum number of motifs of 10, and an optimum motif width of 6-200.

### Chromosomal distribution and Ka/Ks analysis

In order to locate the ClVQ genes, we retrieved their location information from the GFF annotation file from the Coge database. We then used TBtools software to map their distribution on the chromosome [44]. To identify paralogs and orthologs, we ran a BLASTN [45] for the nucleotide sequences of all VQ genes, following the same method described by Blanc & Wolfe [46]. We calculated non-synonymous (ka) and synonymous (ks) substitutions using TBtools [44].

### Analysis of *ClVQ* genes regulatory elements

The 2 kb sequences upstream of the start codon for *ClVQ* genes were obtained in FASTA format from the Coge database. These sequences were then uploaded to PlantCARE (http://bioinformatics.psb.ugent.be/webtools/plantcare/html/) for identification and analysis of Cis-elements.

### RNA extraction and quantitative real-time PCR (qRT-PCR)

RNA from each sample was extracted using the Aidlab plant RNA kit (Aidlab Biotech, Beijing, China) following specific protocols. To ensure quality, the concentration and integrity of all RNAs were assessed using electrophoresis and NanoDrop™ One/OneC (ThermoFisher SClentific, USA). The EF1α gene was used as the reference gene, and gene-specific primers were designed and checked for specificity using Primer Premier 5.0 and TBtools, respectively (Table S8). The first strand of cDNA was synthesized using the Prime Script^TM^RT reagent Kit (TaKaRa, Dalian, China). Real-time PCR was performed on a CFX96^TM^ Real-Time System (BIO-RAD, California, USA) using TB Green Premix Ex Taq II (Tli RNaseH Plus; TaKaRa Biotechnology) with a sample volume of 10 µL. The standard 2^−∆∆CT^ method was used to calculate the relative expression levels of each gene [47].

### Y2H assays

The ClVQ4, ClVQ12 and ClVQ26 CDSs were cloned into the decoy vector pGBKT7, and then transformed into yeast strain Y2HGold (Weidi Biotechnology, shanghai, China) to verify the self-activation. Transformants were screened and verified by SD/-Trp and SD/-Ura/-His/-Trp/-Leu/X-α-Gal. To confirm protein-protein interactions, full-length CDSs of three VQ proteins were cloned into the prey vector pGADT7. Transformants were screened and verified by SD/-Trp/-Leu and SD/-Ura/-His/-Trp/-Leu. Primers used for amplifying these fragments for yeast two-hybrid assays are listed in Supplementary Table S8.

### Statistical and Pearson correlation analysis

Statistical analyses were conducted using Dunnett's two-tailed t test. Mean values and standard deviations (SD) of three replicates were presented, with significant differences relative to controls denoted as ∗*P* ≤ 0.05 and ∗∗*P* ≤ 0.01. Pearson correlation coefficients (PCCs) and *p*-values of stress-induced *ClVQ* gene pairs were obtained and plotted using the R package based on the qRT-PCR results. The coexpression network was constructed using Cytoscape by gathering all gene pairings with PCC values greater than 0.5 and significant at the 0.05 significance level (*P*-value).

## Supplementary Information

**Additional file 1.**

**Additional file 2.**

## Data Availability

The genome sequences of coix were downloaded from Coge database (https://genomevolution.org/coge/OrganismView.pl?dsgid=54616). The genome sequences of *A. thaliana* were downloaded from Phytozome database (https://phytozome-next.jgi.doe.gov/info/Athaliana_TAIR10). The genome sequences of rice were downloaded from Phytozome database (https://phytozome-next.jgi.doe.gov/info/Osativa_v7_0). The genome sequences of maize were downloaded from Phytozome database (https://phytozome-next.jgi.doe.gov/info/Zmays_RefGen_V4). The datasets supporting the results of this article are included in the article and Additional files.
