# Supplementary material for: Genome-wide identification and expression analysis of VQ gene family under abiotic stress in Coix lacryma-jobi L
Source: BMC Plant Biol. 2023 Jun 20;23:327. doi: 10.1186/s12870-023-04294-9 (PMC10280849; doi:10.1186/s12870-023-04294-9)
Supplement: Supplementary file 1 — Additional file 1. [file 12870_2023_4294_MOESM1_ESM.docx]

**Supplementary Figures**


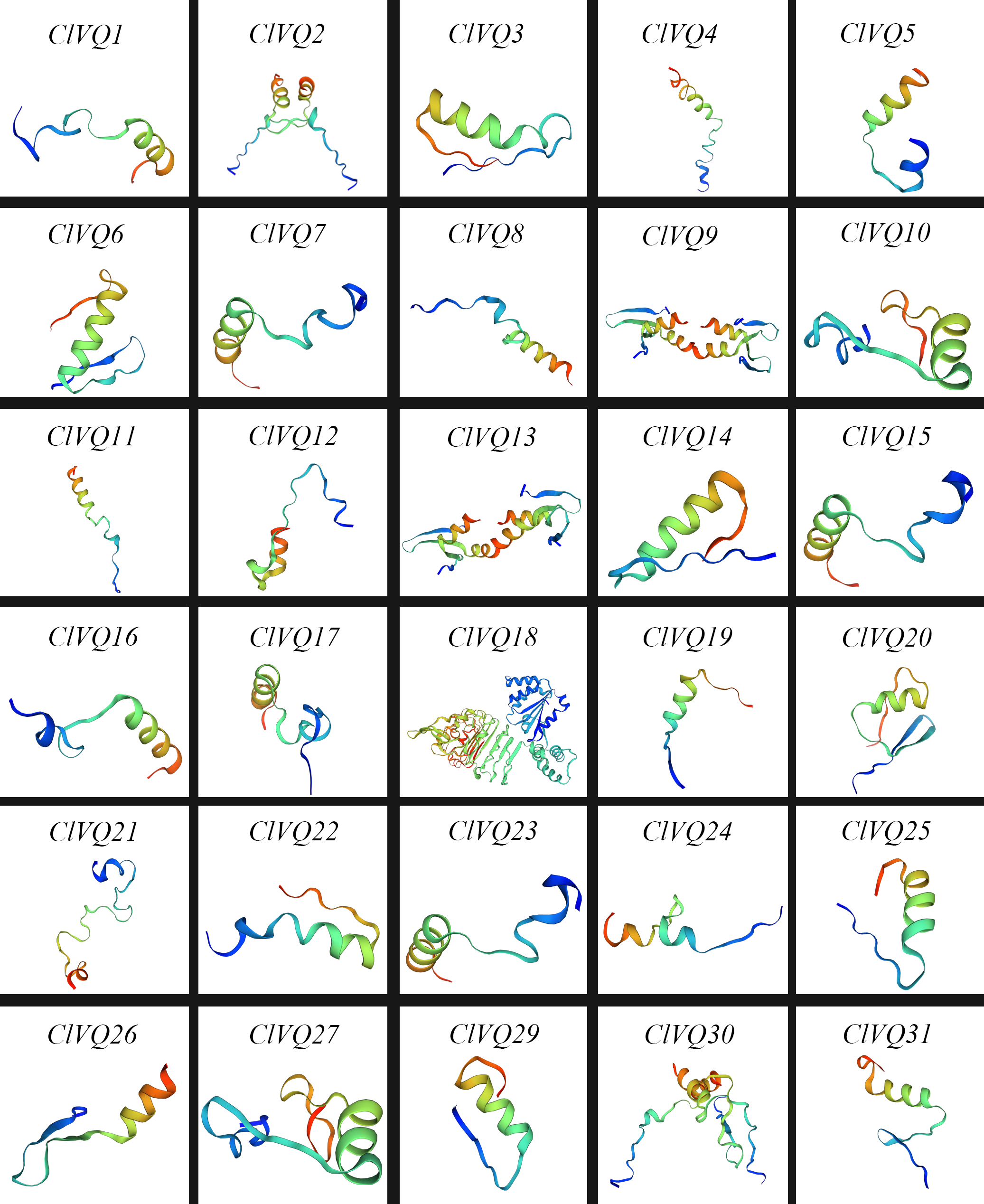


**Fig. S1 Tertiary structures of the ClVQ proteins.** Protein models were obtained using the SWISS-MODEL online server.


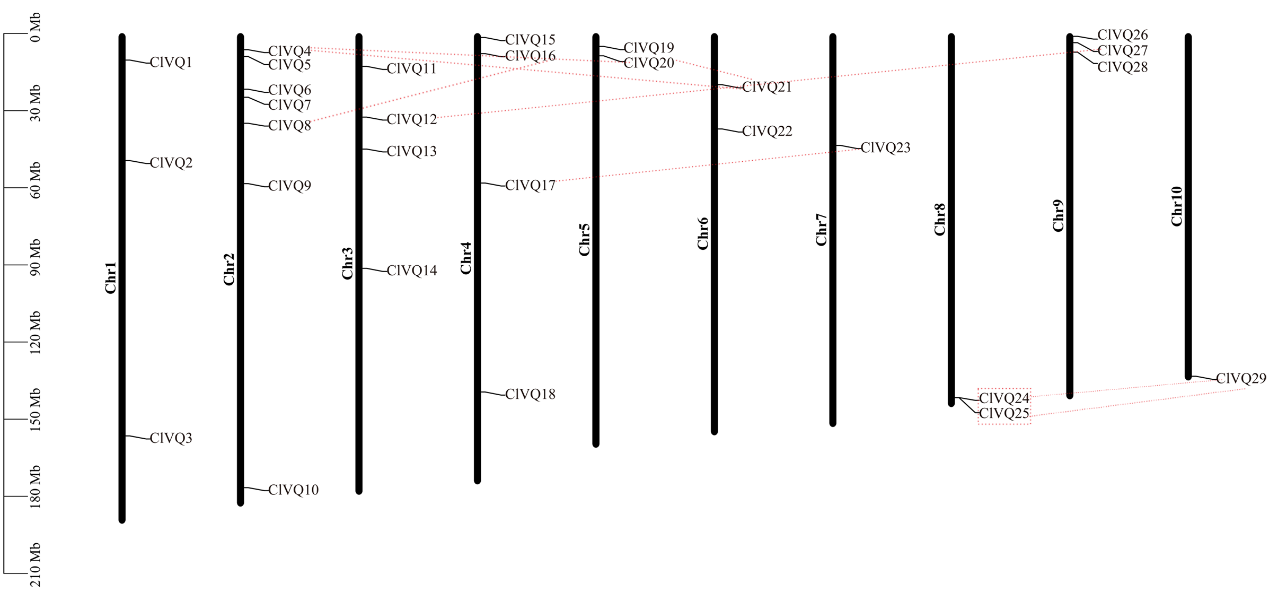


**Fig. S2 Chromosomal location of *VQ* genes in coix.**

The 31 *ClVQ* genes are widely mapped to 10 chromosomes of coix. The paralogous pairs of *ClVQ* gene are connected with red-dotted lines.


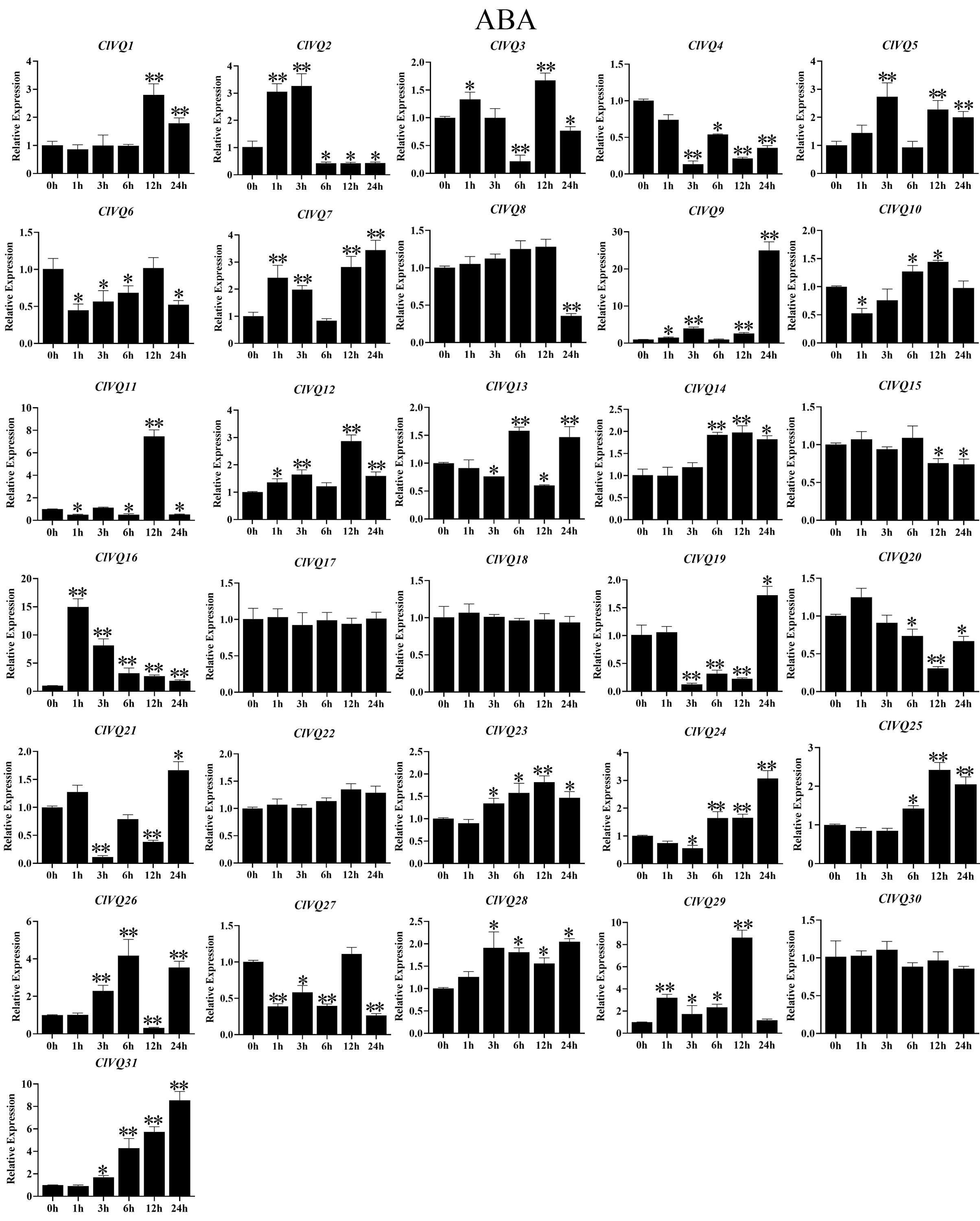


**Fig. S3 Expression analysis of 31 *ClVQ* genes following ABA treatments by qRT-PCR.**

The Y-axis and X-axis indicates relative expression levels and the time courses of stress treatments, respectively. Mean values and standard deviations (SDs) were obtained from three biological and three technical replicates. The error bars indicate standard deviation.


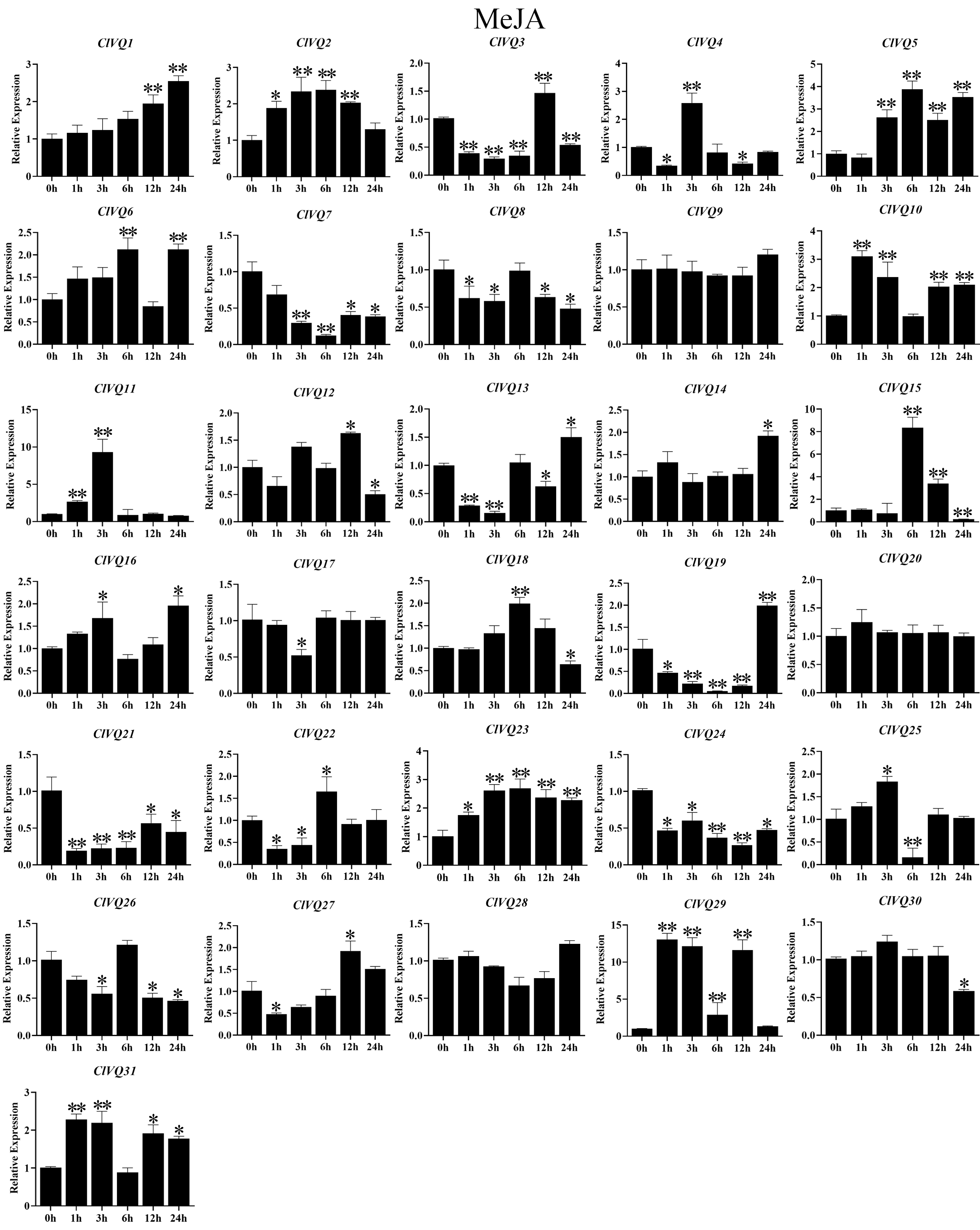


**Fig. S4 Expression analysis of 31 *ClVQ* genes following MeJA treatments by qRT-PCR.**

The Y-axis and X-axis indicates relative expression levels and the time courses of stress treatments, respectively. Mean values and standard deviations (SDs) were obtained from three biological and three technical replicates. The error bars indicate standard deviation.


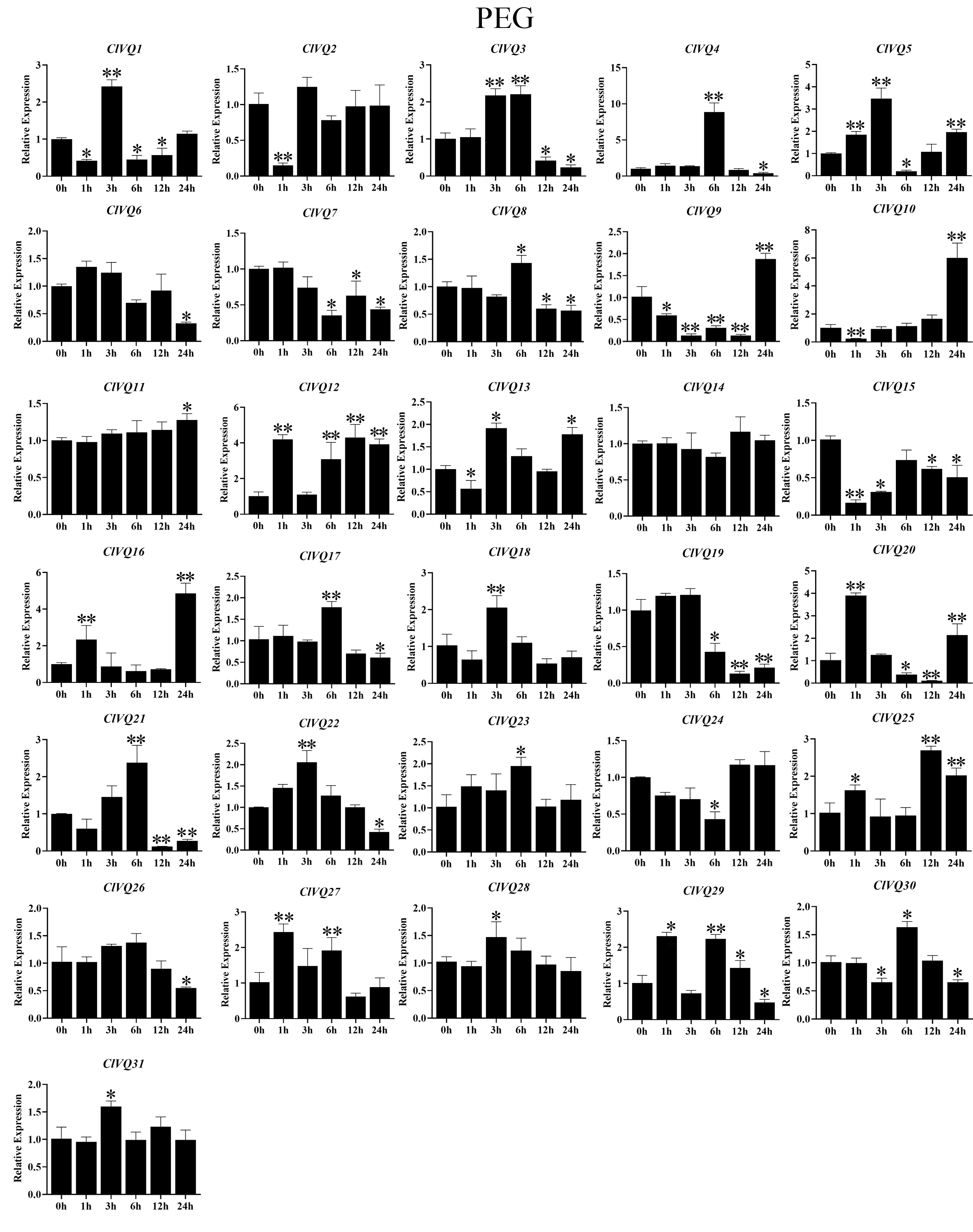


**Fig. S5 Expression analysis of 31 *ClVQ* genes following drought treatments by qRT-PCR.**

The Y-axis and X-axis indicates relative expression levels and the time courses of stress treatments, respectively. Mean values and standard deviations (SDs) were obtained from three biological and three technical replicates. The error bars indicate standard deviation.


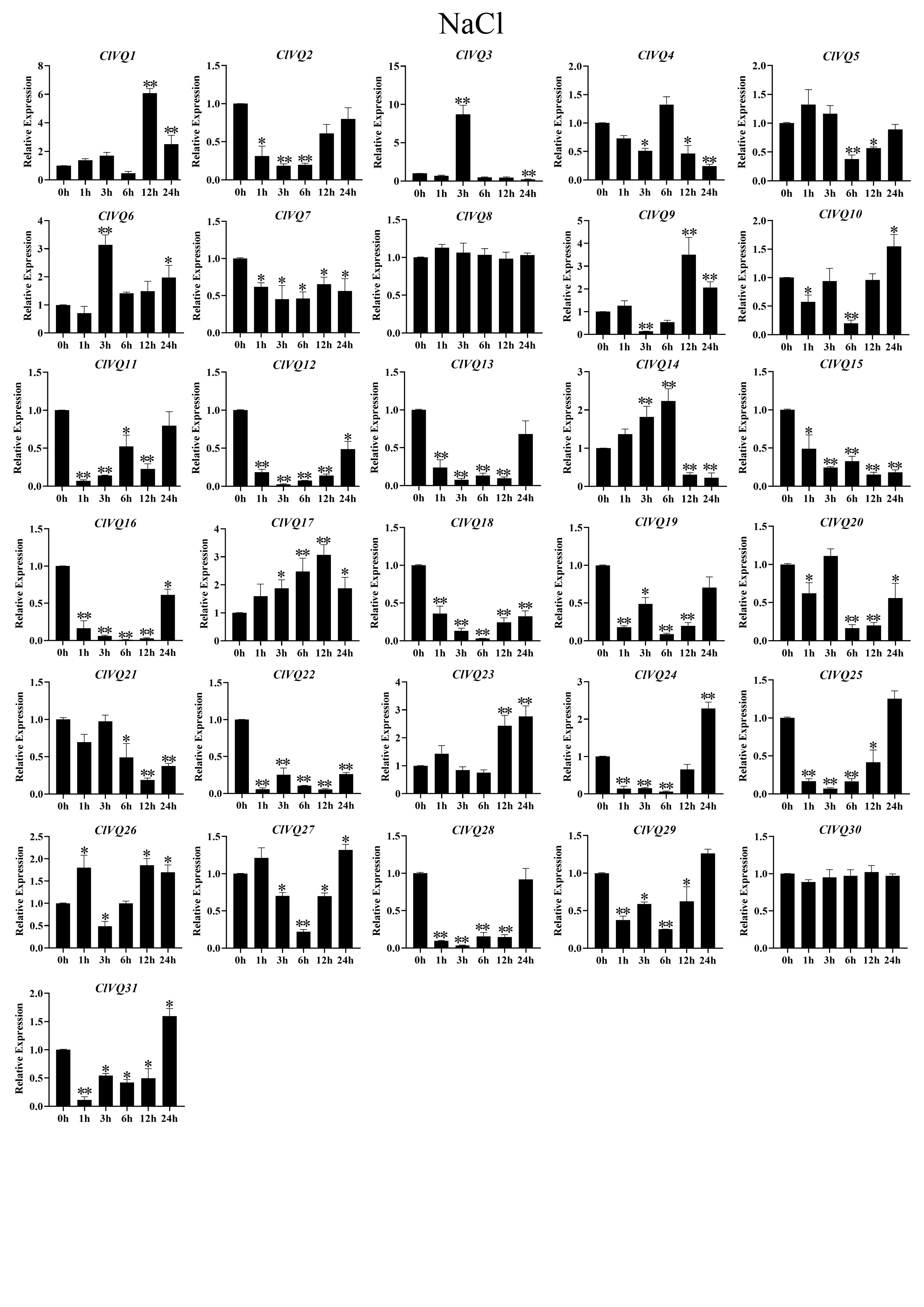


**Fig. S6 Expression analysis of 31 *ClVQ* genes following NaCl treatments by qRT-PCR.**

The Y-axis and X-axis indicates relative expression levels and the time courses of stress treatments, respectively. Mean values and standard deviations (SDs) were obtained from three biological and three technical replicates. The error bars indicate standard deviation.


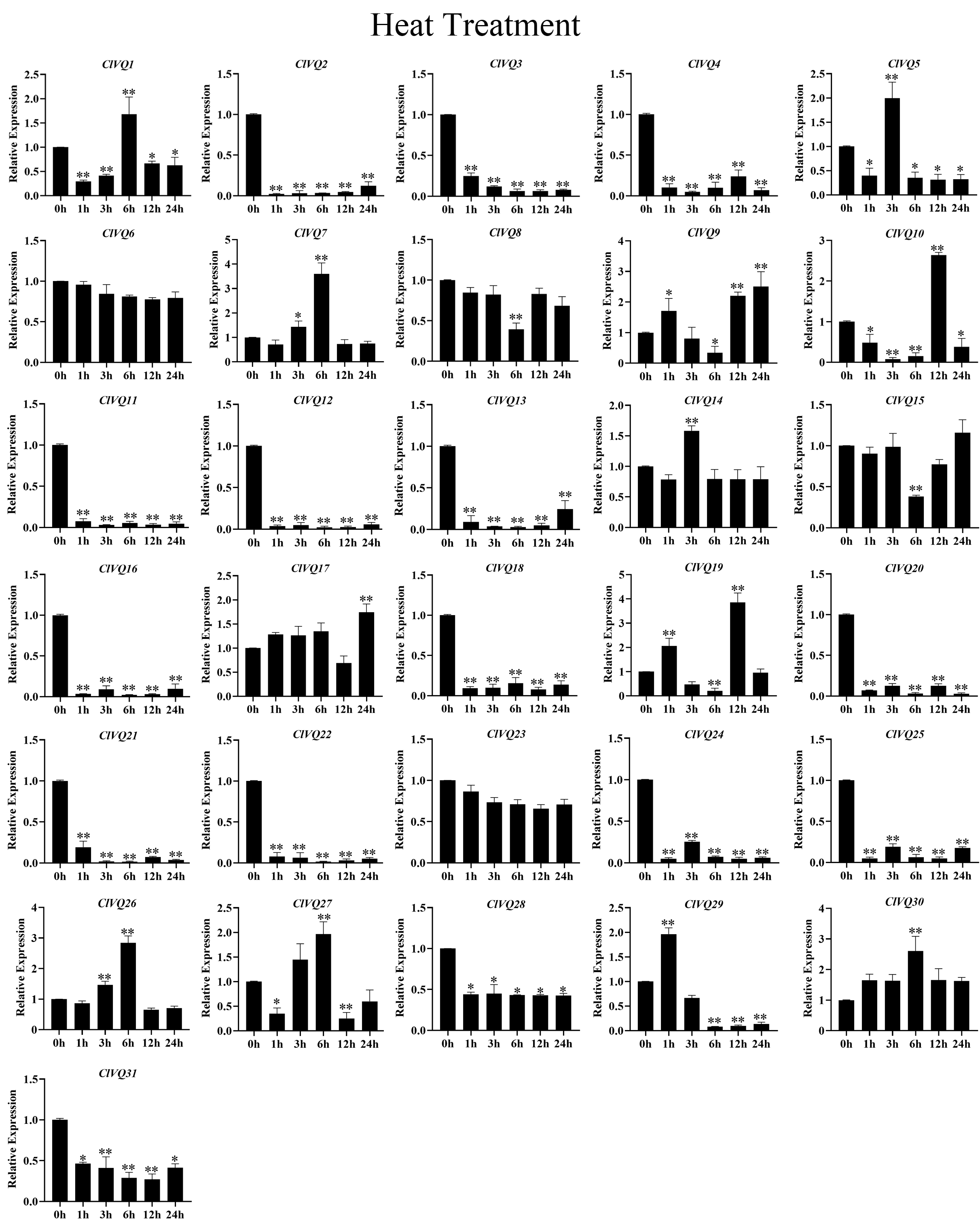


**Fig. S7 Expression analysis of 31 *ClVQ* genes following Heat treatments by qRT-PCR.**

The Y-axis and X-axis indicates relative expression levels and the time courses of stress treatments, respectively. Mean values and standard deviations (SDs) were obtained from three biological and three technical replicates. The error bars indicate standard deviation.
